# Melatonin ameliorates serobiochemical alterations and restores the cardio-nephro diabetic vascular and cellular alterations in streptozotocin-induced diabetic rats

**DOI:** 10.3389/fvets.2023.1089733

**Published:** 2023-03-31

**Authors:** Khalaf F. Alsharif, Ehab Kotb Elmahallawy, Mohamed A. Alblihd, Asmaa A. Hamad, Nani Nasreldin, Walaa Alsanie, Ahmad Majed Aljoudi, Atif Abdulwahab A. Oyouni, Osama M. Al-Amer, Alaa Jameel A. Albarakati, Maha S. Lokman, Ashraf Albrakati, Fatma Abo Zakaib Ali

**Affiliations:** ^1^Department of Clinical Laboratories Sciences, College of Applied Medical Sciences, Taif University, Taif, Saudi Arabia; ^2^High Altitude Research Center, Taif University, Taif, Saudi Arabia; ^3^Department of Zoonotic Diseases, Faculty of Veterinary Medicine, Sohag University, Sohag, Egypt; ^4^Department of Medical Microbiology and Immunology, College of Medicine, Taif University, Taif, Saudi Arabia; ^5^Department of Biology, College of Science, Taif University, Taif, Saudi Arabia; ^6^Department of Pathology and Clinical Pathology, Faculty of Veterinary Medicine, New Valley University, El-Kharga, Egypt; ^7^Hera General Hospital, Makkah, Saudi Arabia; ^8^Department of Biology, Faculty of Sciences, University of Tabuk, Tabuk, Saudi Arabia; ^9^Genome and Biotechnology Unit, Faculty of Sciences, University of Tabuk, Tabuk, Saudi Arabia; ^10^Department of Medical Laboratory Technology, Faculty of Applied Medical Sciences, University of Tabuk, Tabuk, Saudi Arabia; ^11^Department of Surgery, College of Medicine, Umm Al Qura University, Makkah, Saudi Arabia; ^12^Department of Biology, College of Science and Humanities in Al-Kharj, Prince Sattam Bin Abdulaziz University, Al-Kharj, Saudi Arabia; ^13^Department of Zoology and Entomology, Faculty of Science, Helwan University, Cairo, Egypt; ^14^Department of Human Anatomy, College of Medicine, Taif University, Taif, Saudi Arabia; ^15^Department of Pathology and Clinical Pathology, Faculty of Veterinary Medicine, Sohag University, Sohag, Egypt

**Keywords:** melatonin, TNO, endothelin-1, H-FABP, heart, kidney

## Abstract

Melatonin possesses a wide range of pharmacological activities, including antidiabetic properties. Diabetes mellitus (DM) induces several physiopathological changes in body organs, which could be observed lately after systemic failure. In the current study, we aimed to investigate the serobiochemical changes and the histopathological picture in the diabetic heart and the kidney early before chronic complications and highlight the association between hyperglycemia, glomerular alterations, and cardiovascular changes. In addition, the role of melatonin in the treatment of cardio-nephro diabetic vascular and cellular adverse changes in streptozotocin-induced diabetic rats was also studied. A total of 40 mature Wistar albino rats were distributed into five groups; (1) control untreated rats, (2) diabetic mellitus untreated (DM) rats, in which DM was induced by the injection of streptozotocin (STZ), (3) control melatonin-treated (MLT), (4) melatonin-treated diabetic (DM + MLT) rats, in which melatonin was injected (10 mg/kg/day, i.p.) for 4 weeks, and (5) insulin-treated diabetic (DM + INS) rats. The serum biochemical analysis of diabetic STZ rats showed a significant (*P* < 0.05) increase in the concentrations of blood glucose, total oxidative capacity (TOC), CK-MB, endothelin-1, myoglobin, H-FABP, ALT, AST, urea, and creatinine as compared to control rats. In contrast, there was a significant (*P* < 0.05) decrease in serum concentration of insulin, total antioxidative capacity (TAC), total nitric oxide (TNO), and total protein level in DM rats vs. the control rats. Significant improvement in the serobiochemical parameters was noticed in both (DM + MLT) and (DM + INS) groups as compared with (DM) rats. The histological examination of the DM group revealed a disorder of myofibers, cardiomyocyte nuclei, and an increase in connective tissue deposits in between cardiac tissues. Severe congestion and dilation of blood capillaries between cardiac muscle fibers were also observed. The nephropathic changes in DM rats revealed various deteriorations in glomeruli and renal tubular cells of the same group. In addition, vascular alterations in the arcuate artery at the corticomedullary junction and interstitial congestion take place. Melatonin administration repaired all these histopathological alterations to near-control levels. The study concluded that melatonin could be an effective therapeutic molecule for restoring serobiochemical and tissue histopathological alterations during diabetes mellitus.

## 1. Introduction

Diabetes mellitus (DM) is considered one of the most typical critical metabolic and long-term disorders with a steady increase in its prevalence (1). It is characterized by elevated blood glucose levels that might result from an insufficient pancreatic insulin production or an ineffective body insulin utilization with subsequent microvascular and macrovascular complications (2, 3). It is evident that diabetes is associated with several cardiovascular complications, including stroke, myocardial infarction (MI), congestive heart failure (CHF), coronary artery disease (CAD), and peripheral vascular disease (PVD) (3). Taking into account the cardiovascular complications, diabetic cardiomyopathy (DCM) is a prominent consequence of both type I and type II diabetes mellitus, distinguished by functional and structural alterations in the myocardium of diabetic individuals. The main mechanisms of DCM tend to be closely associated with hyperglycemia and advanced generation of glycation end-products, subsequent endothelial dysfunction, increased inflammation, oxidative stress, and fibrosis (4). Furthermore, the alterations in the microvasculature and glomerular endothelial changes constitute the main cause of diabetic nephropathy and cardiopathy (5). Revising the available literature, the STZ-induced diabetic rats are regarded as ideal laboratory animals for the investigation of the fundamental processes underlying diabetic cardiovascular problems and their time-dependent development (6, 7).

Melatonin (5-methoxy-N-acetyltryptamine; MLT) is an indoleamine that is mainly secreted by the pineal gland, in addition to many other organs (8). This indoleamine has strong antioxidant properties besides regulating the daily rhythm in the plasma, pancreas, and pineal gland (9). The previous literature revealed that low MLT concentrations in diabetes are accountable for altered diurnal rhythms and impaired antioxidative ability of tissues (9). Furthermore, diabetic rats and humans have lower plasma levels of MLT than healthy rats and people (10). In addition to its strong antioxidant properties, MLT performs a variety of biological activities, including antiparasitic, anti-apoptotic, and anti-inflammatory actions in a variety of conditions, including obesity and improving fertility (11–17). In rats with myocardial ischemic-reperfusion damage, it was recently shown that the biochemical indicators of cardiac function decreased following treatment with MLT (18), which may be attributable to its antioxidant activities in addition to the activation of apoptosis in cardiomyocytes (19). In addition, MLT's antioxidant properties protect left ventricular dysfunction after myocardial ischemia (20–22). Several previous pieces of evidence suggested that melatonin treatment could improve diabetic nephropathy (DN) and protect the function of the kidney by reducing urinary excretion or protecting podocytes (23, 24). Melatonin has therapeutic potential in DN by preventing inflammation, and fibrosis reduces inflammation and fibrosis in DN through TLR4, i.e., the TGF-β1/Smad3 signaling pathway (25). Reviewing the previous studies, several articles explored the potential role of melatonin in the restoration of serobiochemical and cardio-nephro diabetic alterations. However, limited information is available on the potential influence of melatonin on the serobiochemical changes and the cardio-nephro diabetic vascular and cellular alterations early before the onset of chronic complications in streptozotocin-treated rats. Evidently, the present study was performed to determine the impact of melatonin supplementation on diabetic rats caused by streptozotocin, followed by exploring the serobiochemical alteration combined with describing the histopathological changes in the heart and the kidney.

## 2. Materials and methods

### 2.1. Materials

#### 2.1.1. Drugs and chemicals

A powder form of streptozotocin, citric acid monohydrate and trisodium citrate dihydrate, was obtained from Sigma-Aldrich Company (St. Louis, MO, USA). The N-acetyl-5-methoxytryptamine (Melatonin) was purchased from Fagron (Cat# 420 33457-24, Fagron, Nazareth, Belgium). Insulin vials (Mixtard 30, Novo Nordisk, Denmark) were purchased from a local pharmacy. Industrial serobiochemical kits (Human, Wiesbaden, Hessen, Germany), Spine React (Spain), and Diagnosticum Zrt (Budapest, Hungary) commercial kits were purchased. Biochemical parameters were measured by spectrophotometric techniques (5010 V5+, semi-automatic photometer, RIELE, Germany). The rat insulin enzyme immunoassay kit (Société de Pharmacologie et d'Immunologie—BIO, France), Rat Endothelin 1 (EDN1) ELISA kit (Kamiya Biomedical Company, WA, USA), Rat H-FABP ELISA kit, Rat Myoglobin ELISA kit (Kamiya Biomedical Company, WA, USA), and ELISA kit (Labor Diagnostika Nord GmbH & Co. KG, Germany) were used for oxidative parameters. A nitrate/nitrite was measured by colorimetric assay kit (Cayman Chemical Company, USA) and was employed using microplate reader (ChroMate, Model 4300 microplate reader, FL, USA).

#### 2.1.2. Animals

A total of 40 mature male Wistar albino rats, aged 6 weeks and of body weight range 165–200 g, were used in the present study. The rats were bought from the experimental animal house of the Faculty of Medicine, Sohag University. Specific pathogen-free, hygienic, stainless-steel cages were employed to house the animals with a 12 h light/12 h dark cycle at a humidity of 50:55% and a temperature of 23 ± 2°C. During the course of the experiment, the rats received a standard pellet diet along with unlimited access to water (*ad libitum*). To maintain a clean environment, the bedding was often replaced. Animals were left for 1 week for acclimatization before starting the experiment.

#### 2.1.3. Induction of diabetes

Before this step, the rats were fasted overnight (12 h before diabetic induction). Single intraperitoneal injection of freshly produced streptozotocin (45 mg/kg body weight) in 0.1 M cold citrate buffer caused diabetes (pH 4.5) (26, 27). After the injection, the rats were given free access to food and water, as well as a 15% glucose solution to drink overnight to prevent hypoglycemia. Measurements of blood glucose levels and the onset of diabetes were assessed from 3 to 6 days after STZ treatment, following the protocols described elsewhere (28–31). A glucometer (On Call Plus, ACON Laboratories, Germany) measured the blood sugar levels, which were used to identify diabetic animals. The only rats with blood glucose levels of 250 mg/dl or above following STZ administration were declared diabetic and included in the research. Polydipsia and polyuria in animals were considered as indicators of diabetes.

#### 2.1.4. Experimental design

The rats were kept under close observation for 1 week before the experimental trial. Fecal samples from each group were tested by a concentration technique using floatation and sedimentation during this week. To prevent any parasite infestation prior to the experiment, samples from the sediment and supernatant fluid after centrifugation were tested separately. The rats were divided into five distinct isolated groups, each consisting of eight rats per group, after this week of acclimatization; (1): The rats in the normal control group only received water and standard rat chow; (2) the diabetic mellitus (DM) untreated group did not receive anything; (3) the melatonin-treated (MLT) group were administered 10 mg/kg/day melatonin *via* IP injection (21, 22) for 4 weeks. Melatonin was prepared by dissolving in DMSO (1% w/v) immediately before injection (26, 29); (4) rats in the DM + melatonin-treated group (DM + MLT) received the same melatonin treatment as that given to the MLT group after STZ treatment (the same as the DM group); and (5) rats in the DM + insulin (DM + INS) group were administered insulin subcutaneously (10 units/kg b.w./day) (32) daily for 4 weeks. After 12 h of the last treatment, all rats were sacrificed, and blood and tissue samples were taken.

### 2.2. Methods

#### 2.2.1. Blood sampling

At the end of the experiment, blood samples were collected from the retro-orbital venus plexus (by using a microhematocrit tube) of each rat in plain vacutainer tubes, which were then kept in an inclined position for 20 min at room temperature. Then, these tubes were kept in the refrigerator for a complete retraction of the blood clot. After that, the blood samples were centrifuged for 10 min at 3,000 rpm (1,107×g) to extract the clear serum, which was then collected and stored at −80°C in Eppendorf tubes until biochemical analysis.

#### 2.2.2. Effects of melatonin on the hepatic function parameters in streptozotocin-induced diabetic rats

In this step, serum biochemical parameters, including alanine aminotransferase (ALT), aspartate aminotransferase (AST), total protein, albumin/globulin (A/G) ratio, albumin, and glucose, were measured. The quantitative measurement of blood glucose (BG) was performed using the glucose oxidase enzymatic technique (33), while the kinetic method was used for the determination of both ALT and AST (33, 34). The colorimetric test (Biuret technique) was employed for the quantitative measurement of total proteins (TPs) (35, 36), and albumin was measured by the bromocresol green method (37). The abovementioned measured parameters were counted using industrial kits (Human, Wiesbaden, Germany), while glucose was quantified using Spine/React (Spain) (33). As specified by the manufacturer, all of the aforementioned blood biochemical parameters were measured by spectrophotometric techniques (5010 V5+, semi-automatic photometer, RIELE, Germany). The concentration of globulin and the albumin/globulin (A/G) ratio (38) were calculated. Insulin concentration in serum was determined using the rat insulin enzyme immunoassay kit (Société de Pharmacologie et d'Immunologie—BIO, France) according to the manufacturer's instructions (39).

#### 2.2.3. Effects of melatonin on the cardio- and nephrological function parameters in streptozotocin-induced diabetic rats

In this step, urea was determined using the enzymatic colorimetric assay (40, 41) and a colorimetric (Jaffé reaction) test was used to assess creatinine kinetically (without deproteinization) (41). In addition, creatine kinase-MB (CK-MB) was quantified using Diagnosticum Zrt (Budapest, Hungary) commercial kits. These parameters were measured using spectrophotometric techniques (5010 V5+, semi-automatic photometer, RIELE, Germany) according to the manufacturer instructions. Meanwhile, the determination of endothelin-1 concentration in serum was performed by rat Endothelin 1 (EDN1) ELISA kit (Kamiya Biomedical Company, WA, USA). The quantitative determination of endothelin-1, cardiac fatty acid binding protein (H-FABP), and myoglobin concentration in rat serum was carried out by Rat Endothelin 1 (EDN1) ELISA kit, Rat H-FABP ELISA kit, and Rat Myoglobin ELISA kit (Kamiya Biomedical Company, WA, USA). Colorimetric test was performed for the quantitative determination of the total antioxidative capacity (TAC) in serum and EDTA plasma. ELISA kit (Labor Diagnostika Nord GmbH & Co. KG, Germany), enzymatic test for the measurement of peroxides, total oxidative capacity (TOC) in biological fluids, and ELISA kit (Labor Diagnostika Nord GmbH & Co. KG, Germany) were employed. The total nitric oxide (TNO) measurement was performed using a nitrate/nitrite colorimetric assay kit (Cayman Chemical Company, USA) and the microplate reader (ChroMate, Model 4,300 microplate reader, FL, USA).

#### 2.2.4. Histopathological examination

After the experiment, animals were sacrificed, and tissue samples from the hearts and the kidneys were collected, dissected, and immediately fixed in 10% formalin for 24 h, dehydrated in a series of graded alcohols, cleared in xylene, and blocked in paraffin. Tissue sectioning was done at a thickness of 3–5-μm and stained with hematoxylin and eosin (H&E) (42), and special stains such as Masson's trichrome and periodic acid–Schiff (PAS) (43) for histopathological examination was provided. Heart tissue sections were stained with H&E and Masson's trichrome stains, while kidney tissue sections were stained with H&E and PAS stains. All sections were viewed and imaged using the Olympus CX43 microscope and a microscope-adapted Olympus SC52 camera.

#### 2.2.5. Morphometric study

The analysis of organ histopathology was performed by assigning a score depending on the degree of damage observed in each group in the examined tissue for semiquantitative measurements: 0 = no lesions; 1 = mild (1–25%); 2 = moderate, (26–45%); and 3 = severe (>45%) as described previously (44–46). Furthermore, ImageJ vs. 1.48 software (NIH) was used for measuring the cardiomyocyte widths in H&E-stained sections. Each heart paraffin block was sectioned into five non-overlapping parts and inspected with low-power fields (×400) (47). After system calibration, transverse transnuclear widths of randomly chosen cardiomyocytes were measured. Each sample is represented by the mean of 100 LV cardiomyocytes. Blinded observers measured the quantity of myocardial collagen using semiquantitative morphometric scoring of Masson's trichrome-stained slices. Each sample is represented by the average value of 20 randomly chosen visual fields (400× magnification) of a free LV wall (48, 49). As regards the histomorphometric tissue scoring of the renal tissue, the tissue slices were cut at a thickness of 3–5-μm and stained with hematoxylin and eosin, and periodic acid–Schiff (PAS). Pathologists evaluated the renal tissue specimens. According to the recognized histopathologic categorization for diabetic kidney disease, interstitial lesions, glomerular lesions, vascular lesions, and renal tubular lesions were graded. Diabetes scores were assigned according to previous studies (50, 51). The morphometric measurements were carried out at the Image Analysis Unit, Department of Pathology and Clinical Pathology, Faculty of Veterinary Medicine, Sohag University and Department of Clinical Laboratories Sciences, College of Applied Medical Sciences, Taif University, Taif, Saudi Arabia.

### 2.3. Statistical analysis

The histopathological measurements from the experimental groups were stated as mean ± standard deviation (SD) and estimated using Version 5 of GraphPad Prism (San Diego, California, USA) for analyzing data using the one-way ANOVA and Tukey's *post hoc* multiple-comparisons tests; the statistical significance was considered at a *P*-value of <0.05 (52, 53). As regards the serum biochemical parameters, data were collected and then analyzed using SPSS.28 software. The analysis of variance (ANOVA) was used for comparing the means of the various groups to all serum biochemical variables. Then, multiple comparisons tests (*post hoc* multiple-comparisons LSD, Duncan) were used to evaluate if there were changes in mean differences between the studied groups, which were analyzed using SPSS 28 for the Microsoft Windows operating system. The data are presented as the mean ± SEM. A value of *P* of <0.05 denotes that differences between all groups are statistically significant.

## 3. Results

### 3.1. Melatonin improved the hepatic function parameters in streptozotocin-induced diabetic rats

The serobiochemical results are shown in Tables 1, 2 (*P* < 0.05). In the current study, a significantly elevated ALT was observed in the DM group in comparison with the control group (Table 1). In contrast, the DM + MLT and DM + INS groups showed a significant decrease in ALT as compared with the DM group. The concentrations of AST showed a significant elevation in the DM group compared with the control group. Furthermore, the MLT group revealed a significant increase in AST as compared to the control group (Table 1). While a significant decrease was noted in the AST group in both the DM + MLT and the DM + INS groups as compared with the DM group. As shown in Table 1, there was a significant decrease in total protein in the DM group as compared with the control group. Moreover, there was a significant decrease in total protein in the MLT group as compared with the control group (Table 1). Furthermore, a significant increase was detected in blood glucose level in the DM group as compared to the control group, while a significant reduction in blood glucose level was noticed in the DM + MLT and DM + INS groups as compared with the DM untreated group. On the other hand, serum levels of insulin were significantly decreased in the DM group as compared to the control rats, but significantly elevated insulin was reported in the DM + MLT group and the DM + INS group as compared with the DM group (Table 1).

**Table 1:** Effects of melatonin on the hepatic function parameters among all groups (mean ± SEM).

**Item**	**ALT (U/L)**	**AST (U/L)**	**Total proteins (g/dl)**	**Albumin (g/dl)**	**Globulin (g/dl)**	**A/G ratio**	**Blood glucose (mg/dl)**	**Insulin (ng/ml)**
Control	57.2 ± 3.38^a^	156.4 ± 2.54^a^	7.7 ± 0.2^a^	4.03 ± 0.09^a^	3.69 ± 0.17^a^	1.11 ± 0.05^a^	66.21 ± 3.22^a^	1.23 ± 0.009^a^
DM	138 ± 13.89^b^	191.3 ± 4.32^b^	7.06 ± 0.29^b^	3.72 ± 0.20^ab^	3.33 ± 0.29^ab^	1.17 ± 0.74^ab^	395.2 ± 19.59^b^	0.66 ± 0.012^b^
MLT	44.2 ± 1.96^a^	170.2 ± 3.15^c^	6.6 ± 0.16^bc^	3.29 ± 0.24^b^	3.32 ± 0.30^ab^	1.05 ± 0.16^a^	67.4 ± 1.74^a^	1.22 ± 0.003^a^
DM + MLT	97.7 ± 11.44^c^	175.2 ± 3.81^c^	6.5 ± 0.24^bc^	3.8 ± 0.12^a^	2.67 ± 0.17^b^	1.46 ± 0.08^b^	204.8 ± 13.83^c^	0.82 ± 0.018^c^
DM + INS	64.8 ± 5.52^d^	171.6 ± 5.45^c^	6.7 ± 0.22^bc^	3.83 ± 0.09^a^	2.83 ± 0.14^b^	1.36 ± 0.05^ab^	306.9 ± 16.47^d^	1.06 ± 0.017^d^

a−d Means in the same column with different superscripts are significant at P < 0.05.

ALT, alanine aminotransferase; AST, aspartate aminotransferase; A/G ratio, albumin/globulin ratio.

**Table 2:** Effects of melatonin on the cardiological and nephrological function parameters among all groups (mean ± SEM).

**Item**	**Urea (mg/dl)**	**Creatinine (mg/dl)**	**CK-MB (U/L)**	**TNO (μM/ml)**	**Endothelin-1 (pg/ml)**	**Myogb (ng/ml)**	**H-FABP (ng/ml)**	**TAC (mmol/L)**	**TOC (mmol/L)**
Control	47.56 ± 3.82^a^	0.6 ± 0.04^a^	14.39 ± 0.13^a^	54.73 ± 0.22^a^	0.38 ± 0.003^a^	32.51 ± 0.34^a^	15.18 ± 0.06^a^	1.44 ± 0.01^a^	0.38 ± 0.00^a^
DM	105.72 ± 6.34^b^	0.71 ± 0.02^b^	27.09 ± 0.22^b^	37.09 ± 0.48^b^	0.74 ± 0.011^b^	70.41 ± 0.46^b^	32.26 ± 0.25^b^	0.78 ± 0.01^b^	0.82 ± 0.00^b^
MLT	50.09 ± 2.01^a^	0.69 ± 0.03^bc^	14.16 ± 0.07^a^	54.48 ± 0.25^a^	0.38 ± 0.003^a^	32.36 ± 0.35^a^	15.11 ± 0.08^a^	1.44 ± 0.00^a^	0.38 ± 0.00^a^
DM + MLT	112.76 ± 7.38^b^	0.73 ± 0.01^b^	22.39 ± 0.30^c^	42.81 ± 0.57^c^	0.54 ± 0.015^c^	51.95 ± 0.32^c^	24.09 ± 0.18^c^	0.94 ± 0.00^c^	0.60 ± 0.01^c^
DM + INS	89.18 ± 7.38^c^	0.64 ± 0.03^ac^	19.54 ± 0.18^d^	48.89 ± 0.22^d^	0.43 ± 0.013^d^	44.80 ± 0.34^d^	20.13 ± 0.33^d^	1.15 ± 0.03^d^	0.52 ± 0.00^d^

a−d Means in the same column with different superscripts are significant at a P-value of < 0.05.

CK-MB, creatine kinase-MB; TNO, total nitric oxide; Myogb, myoglobin; H-FABP, cardiac fatty acid-binding protein; TAC, total antioxidative capacity; TOC, total oxidative capacity.

### 3.2. Melatonin improved the cardio- and nephrological functional parameters in streptozotocin-induced diabetic

As depicted in Table 2, there was a significant increase in urea in the DM group as compared to the control group. However, there was a significant decrease in urea in the DM + INS group as compared with the DM group (Table 2). In addition, there was a significant increase in creatinine in the DM group in comparison with the control group (Table 2). Furthermore, there was a significant increase in creatinine between the MLT group and the control group (Table 2). However, there was a significant decrease in creatinine in the DM + INS group compared with the DM group (Table 2). In relation to TOC concentration, which is shown in Table 2, the DM group exhibited a significant increase in its concentration as compared to the control, while a significant reduction in serum concentration of TOC was observed in the DM + MLT and DM + INS groups as compared with the DM untreated group. However, the serum level of TAC significantly decreased in the DM group as compared to the control rats, while significantly elevated TAC concentration was recorded in both the DM + MLT group and the DM + INS group as compared with the DM group (Table 2). In this study, as shown in Table 2, the CK-MB, endothelin-1, myoglobin, and H-FABP serum concentrations were significantly elevated in the DM group as compared to the control group. However, when compared to the DM group, both DM + MLT and DM + INS groups showed a significant reduction in serum concentrations of CK-MB, endothelin-1, myoglobin, and H-FABP (Table 2). On the contrary, the serum concentration of total nitric oxide (TNO) significantly decreased in the DM group compared to the control group; however, its concentration was significantly elevated in the DM + MLT and the DM + INS groups as compared with the DM group (Table 2).

### 3.3. Histopathological assessment

#### 3.3.1. Heart

**Figure 1:**
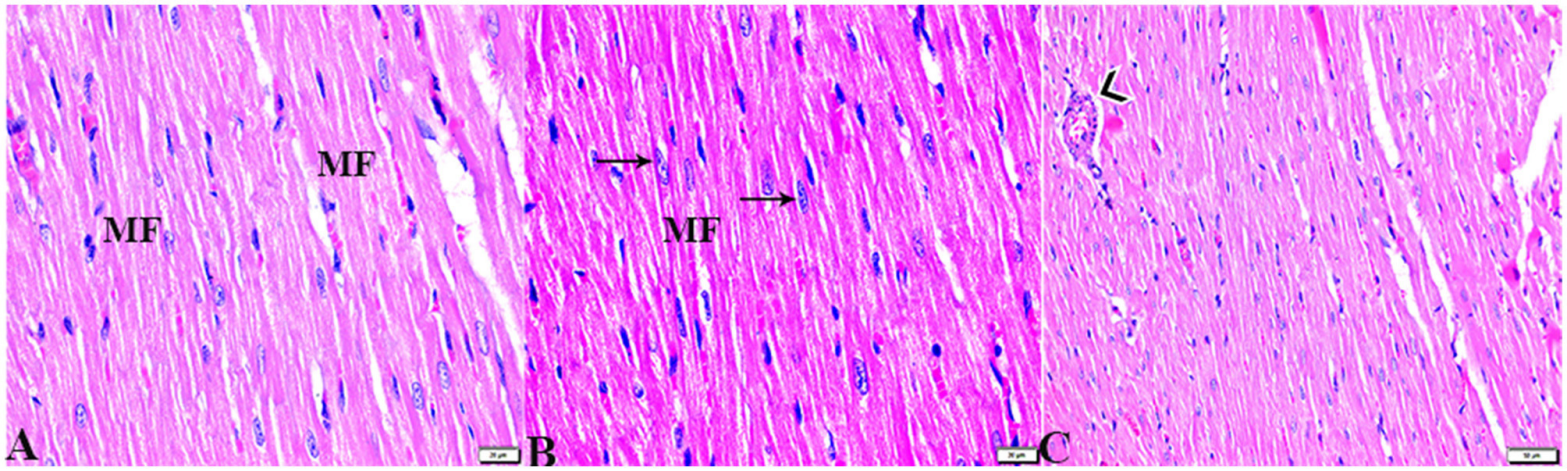
Photomicrograph of longitudinal sections of the left ventricular cardiac tissue sections from control rats (G1): **(A–C)**: branching and anastomosing cardiac muscle fibers (MFs) with an acidophilic sarcoplasm and centrally located cigar-shaped vesicular nuclei (arrows). **(C)**: Normal blood vascular structure (arrowhead). H&E stain. The bar size [**(A, B)** = 20 μm and **(C)** = 50 μm].

**Figure 2:**
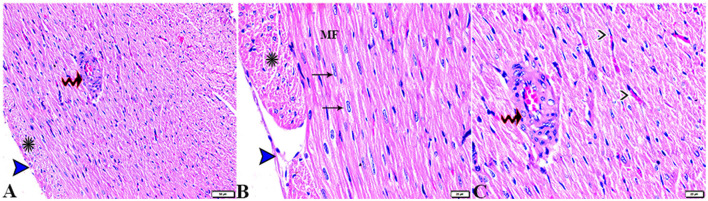
Photomicrograph of ventricular cardiac tissue sections from melatonin control rats (G3): [**(A)** magnified in **(B, C)**]: normal endocardium lined internally with continuous endothelium (blue arrowheads), inner subendothelium containing the impulse-conducting cardiac muscle fibers (Purkinjie fibers) (stars), and branching and anastomosing cardiac muscle fibers (MFs) with an acidophilic sarcoplasm and centrally located cigar-shaped vesicular nuclei (arrows). Normal blood vessel with a normal wall thickening (zigzag arrows). Normal blood capillaries in between cardiac muscle fibers (arrowheads). H&E stain. The bar size [**(A)** = 50 μm and **(B, C)** = 20 μm].

**Figure 3:**
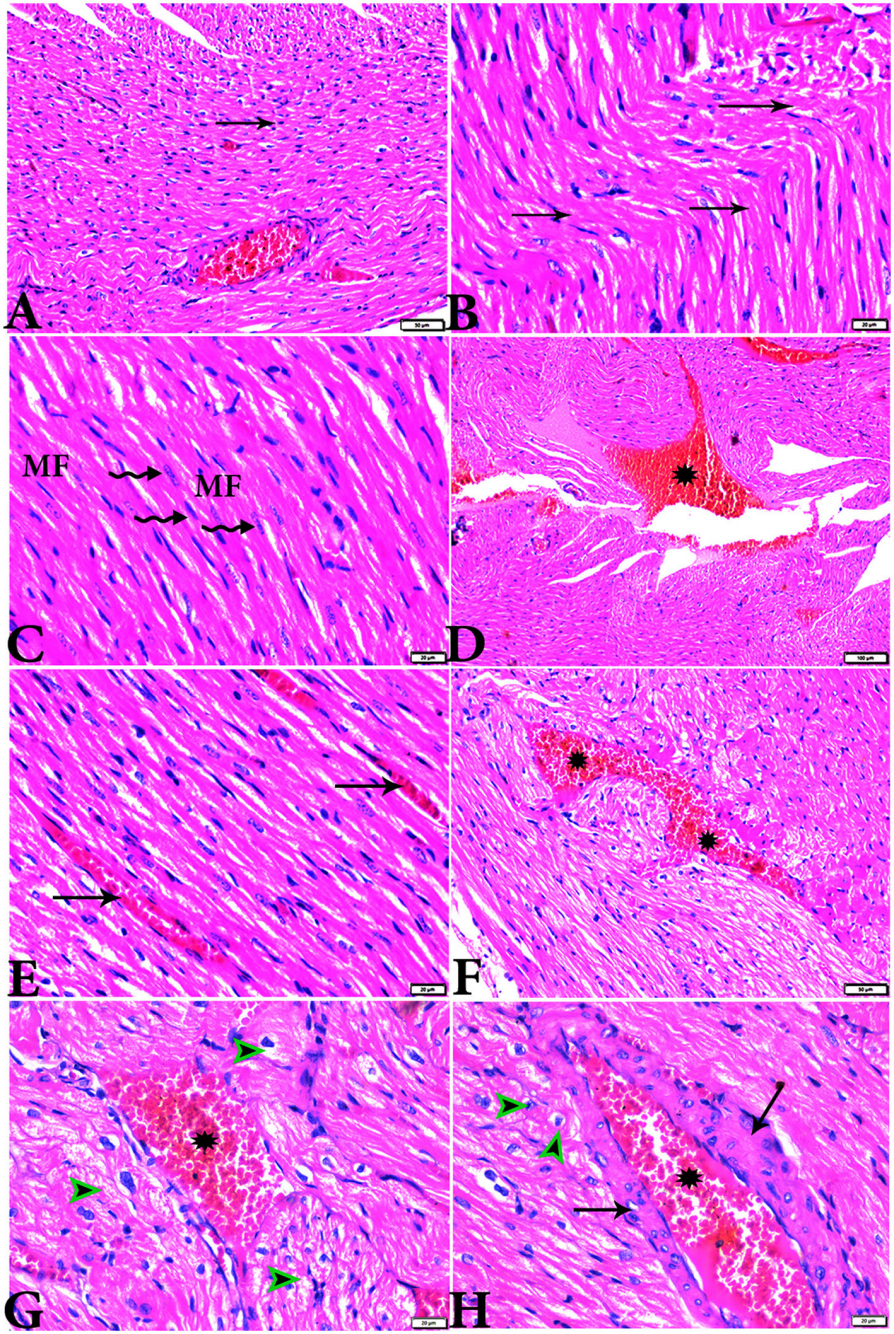
Photomicrograph of ventricular cardiac tissue sections from diabetic untreated rats (G2): **(A, B)**: deformation in sizes with a disarrayed pattern of cardiac muscle fibers (arrows), **(C)**: nuclear changes (zigzag arrows) in cardiac myocytes (MFs), **(D)**: severe congestion (star), **(E)**: congested and dilated capillaries in between cardiac muscle fibers (arrows), **(F, G)**: severe congestion in the blood vessels (stars) surrounded by vacuolated cells (arrowheads), and **(H)**: congested blood vessel (star) with a thick hyalinized wall (arrows), surrounded by vacuolated cells (arrowheads). H&E stain. The bar size [**(A, F)** = 50 μm, **(B, C, E, G, H)** = 20 μm, and **(D)** = 100 μm].

**Figure 4:**
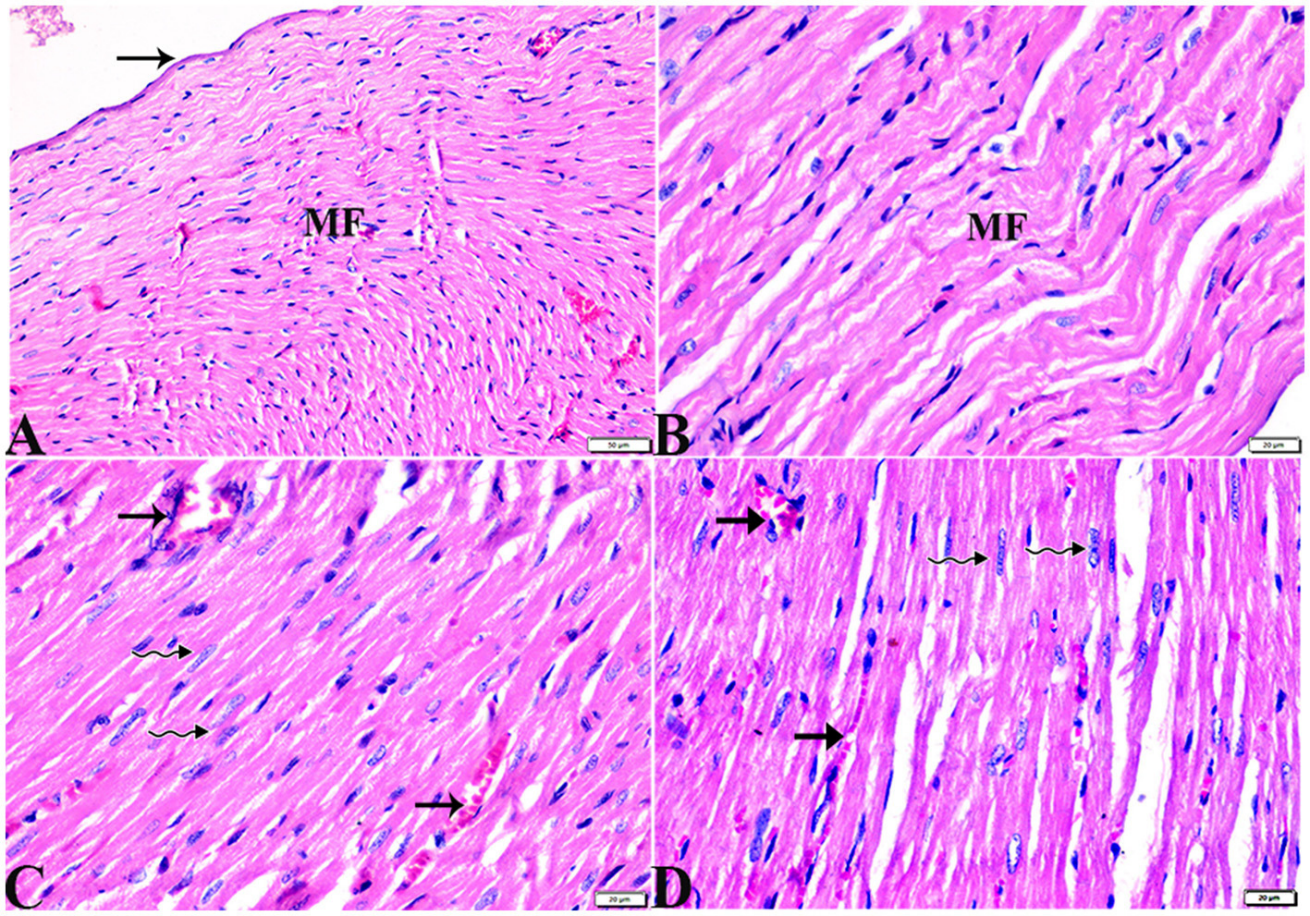
Photomicrograph of cardiac tissue sections from diabetic rats treated with melatonin (G4): **(A)**: normal endocardium lined internally with continuous endothelium (arrow), **(A, B)**: a marked improvement in the orientation and size of cardiac myofibers (MF), **(C, D)**: cardiac muscle fibers (MFs) showed a normal acidophilic sarcoplasm and centrally located cigar-shaped vesicular nuclei (zigzag arrows). Mild congested capillaries in between cardiac muscle fibers (arrows). H&E stain. The bar size [**(A)** = 50 μm, **(B–D)** = 20 μm].

**Figure 5:**
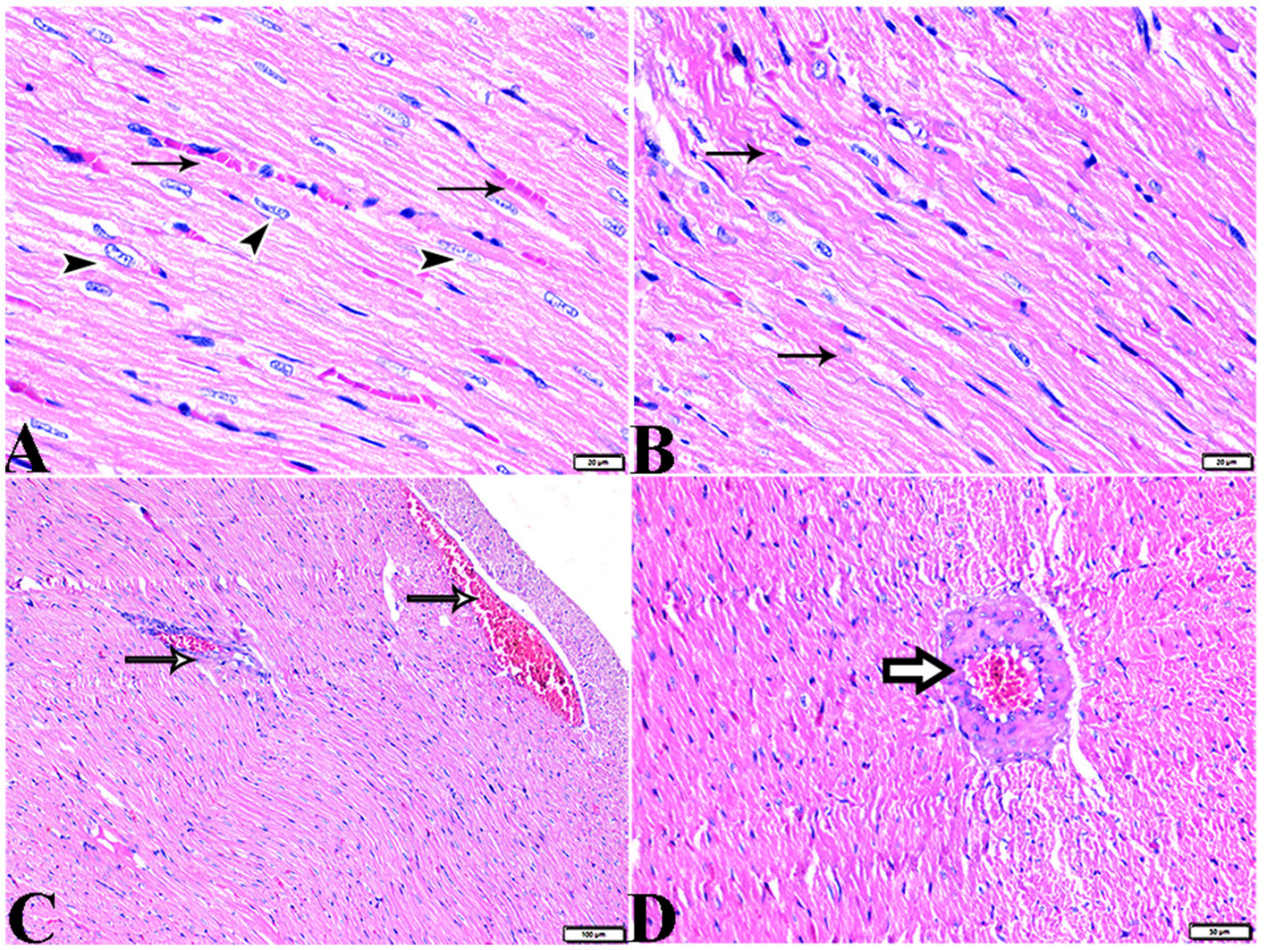
Photomicrograph of cardiac tissue sections from diabetic rats treated with insulin (G5): **(A)**: cardiac muscle fibers with more or less normal acidophilic sarcoplasm and centrally located cigar-shaped vesicular nuclei (arrowheads), mild congested capillaries in between cardiac muscle fibers (arrows), **(B)**: improvement in the orientation and size of cardiac myofibers (arrows), **(C)**: congestion in blood vessels (arrows), and **(D)**: thick vascular wall. H&E stain. The bar size [**(A, B)** = 20 μm, **(C)** = 100, and **(D)** = 50 μm].

**Figure 6:**
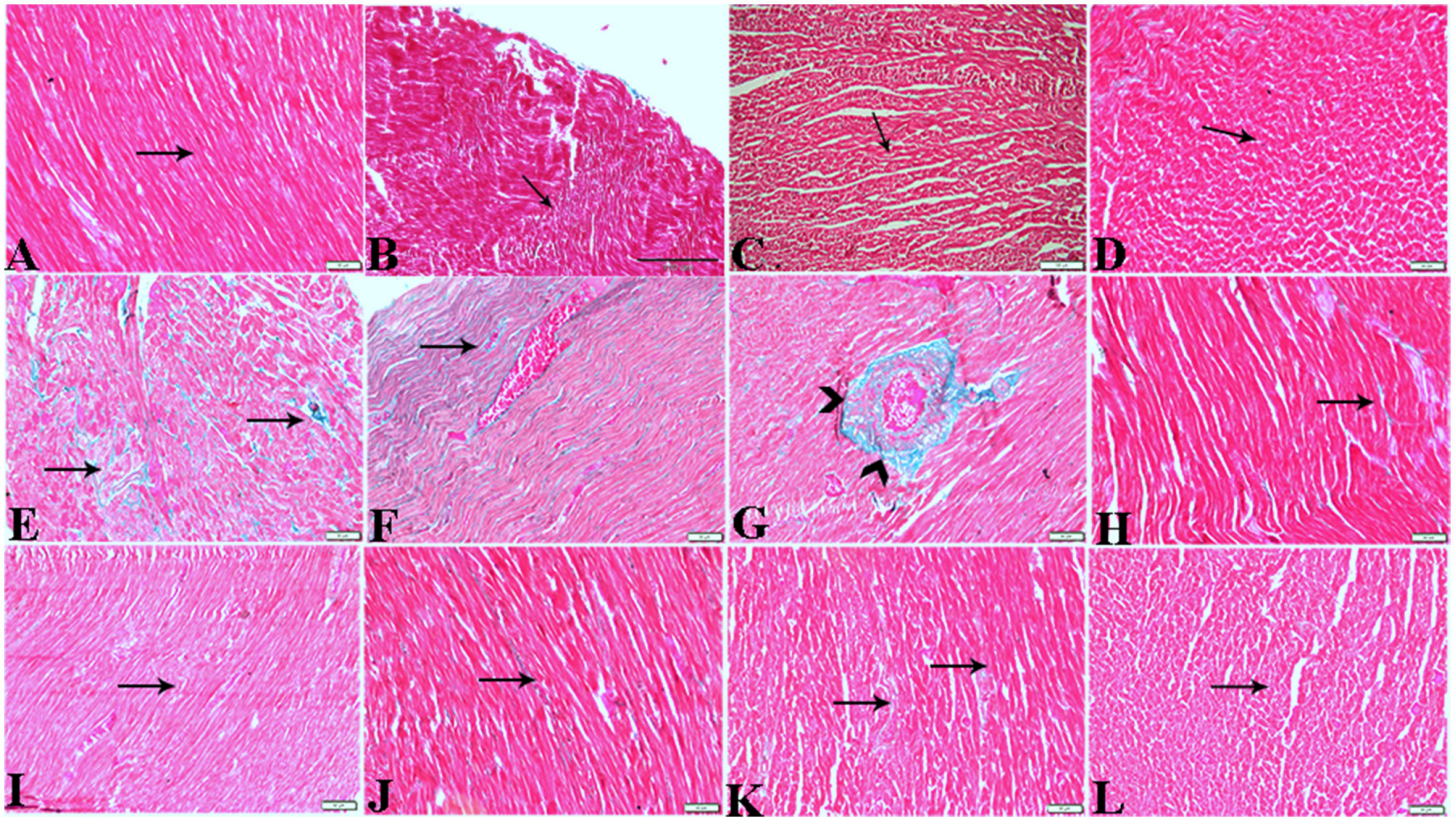
Photomicrograph of cardiac tissue sections from the experimental groups stained with Masson's trichrome stain showed **(A, B)**: cardiac tissue sections from control rats, **(C, D)**: cardiac tissue sections from MT control rats showed normal fine threads of collagenous fibers between cardiac myofibers (arrows), **(E–G)**: cardiac tissue sections from diabetic untreated rats, **(E, F)**: showed dense collagenous fibers between cardiac myofibers (arrows) and thick fibroses in a vascular wall [**(G)**, arrowhead], **(H–J)**: cardiac tissue sections from diabetic rats treated with melatonin showed fine threads of collagenous fibers between cardiac myofibers (arrows), **(K, L)**: cardiac tissue sections from diabetic rats treated with insulin showed fine threads of collagenous fibers between cardiac myofibers (arrows). The bar size [**(A, D–L)** = 50 μm, **(B, C)** = 100 μm].

**Figure 7:**
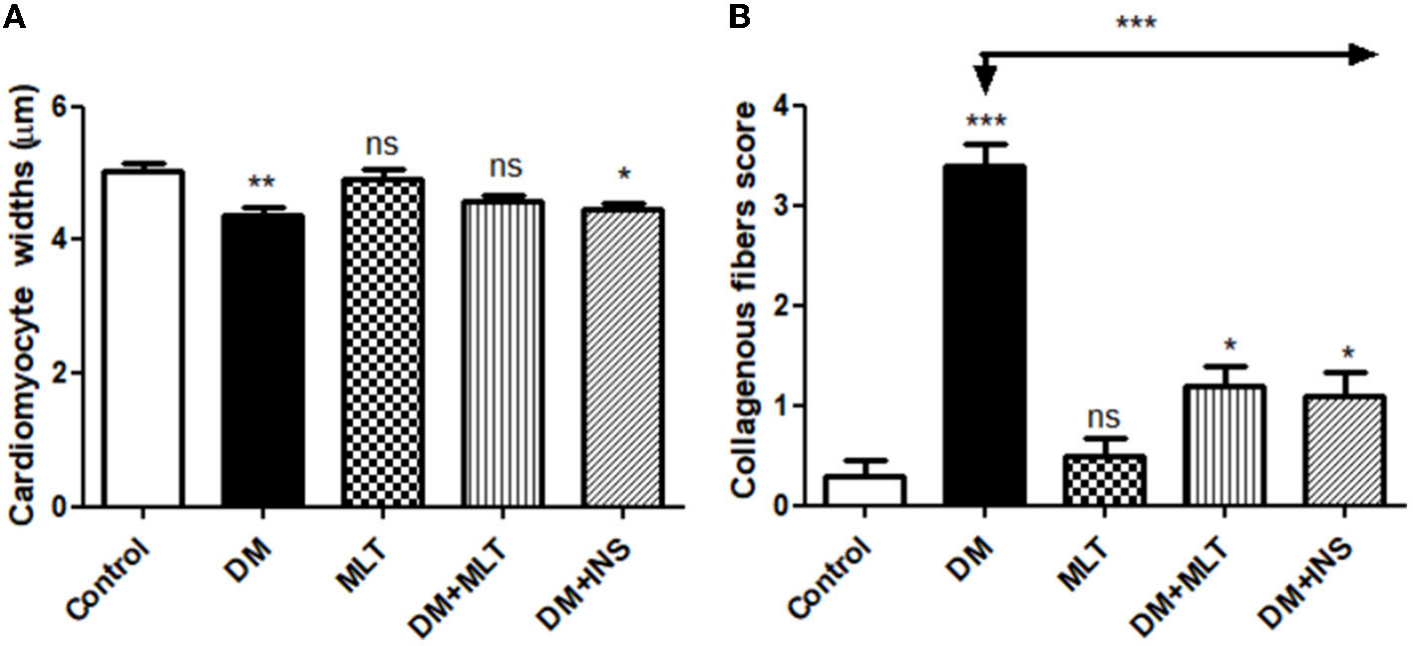
Histomorphometric graph showing quantitative and semiquantitative measurements of lesion scores recorded in cardiac tissue sections among the experimental groups: **(A)**: cardiomyocyte widths and **(B)**: collagenous fibrous score. Data are expressed as means ± standard deviations. Significant differences vs. the control group are marked by different asterisks through one-way ANOVA with Tukey's *post ho*c test: *p ≤ 0.05, **p ≤ 0.01, ***p ≤ 0.001.

The microscopic examination of the cardiac tissue section from the negative control group (Figure 1) and the MLT group (Figure 2) revealed the histological architecture of the endocardium, which is lined internally with continuous endothelium and an inner subendothelium containing the impulse-conducting cardiac muscle fibers (Purkinjie fibers) (Figures 2A, B). The typical myocardium had anastomosing muscle fibers with cigar-shaped vesicular nuclei in the center, longitudinally striated branching, and properly distributed cardiac myofibers (Figures 1, 2). However, sections taken from the DM group showed several pathological findings. Sizes and shapes of the cardiomyocytes in this group exhibited distortion. In addition, cardiac myofibers in the DM group were shown to be disordered (Figures 3A–C), and severely congested and dilated capillaries between cardiac muscle fibers were also observed (Figures 3D, E). In addition, some congested blood vessels showed thick hyalinized walls surrounded by vacuolated cells in the same group (Figures 3F–H). Cardiac tissue sections from diabetic rats treated with melatonin DM + MLT demonstrated a marked enhancement in the histological structure of the endocardium and in the orientation and size of cardiac myofibers (Figures 4A, B). This enhancement appeared with centrally located cigar-shaped vesicular nuclei and normal acidophilic sarcoplasm (Figures 4C, D). Vascular congestion in this group (DM + MLT) was mild as compared with the DM group. Cardiac tissue sections from diabetic rats treated with insulin (DM + INS) showed an improvement in the orientation and size of cardiac myofibers with more or less normal acidophilic sarcoplasm and centrally located cigar-shaped vesicular nuclei (Figures 5A, B), and mild congested thick vascular walls were also noticed (Figures 5C, D). The heart sections stained with Masson's trichrome from the control (Figures 6A, B) and MLT-treated rats (Figures 6C, D) showed sparse and typical collagen fiber spreading, represented by a thin interstitial fibrous connective tissue. Untreated DM rats showed a striking increase in the deposition of collagen fibers in the interstitial region of the heart and around the blood vessels (Figures 6E–G). Similar to insulin therapy, treatment with MLT significantly reduced the fibrosis in the hearts of DM + MLT (Figures 6H–L). The histomorphometric measurements revealed a significant (*P* < 0.05) reduction in the width of cardiomyocytes in DM animals as compared with the experimental groups (Figure 5). However, the DM + MLT group showed a non-significant (*P* < 0.05) change compared with the control group and a significant (*P* < 0.05) change compared with the DM untreated group (Figure 7A). In the cardiac sections of the standard control and MLT rats, there was a fine collagen fiber normal distribution with a thin fibrous interstitial connective tissue. In DM animals, the interstitial myocardial tissue demonstrated a considerable rise in collagen fiber deposition. The treatment with MT significantly reduced fibrosis in the hearts of diabetic rats to the same extent as insulin (Figure 7B).

#### 3.3.2. Kidney

**Figure 8:**
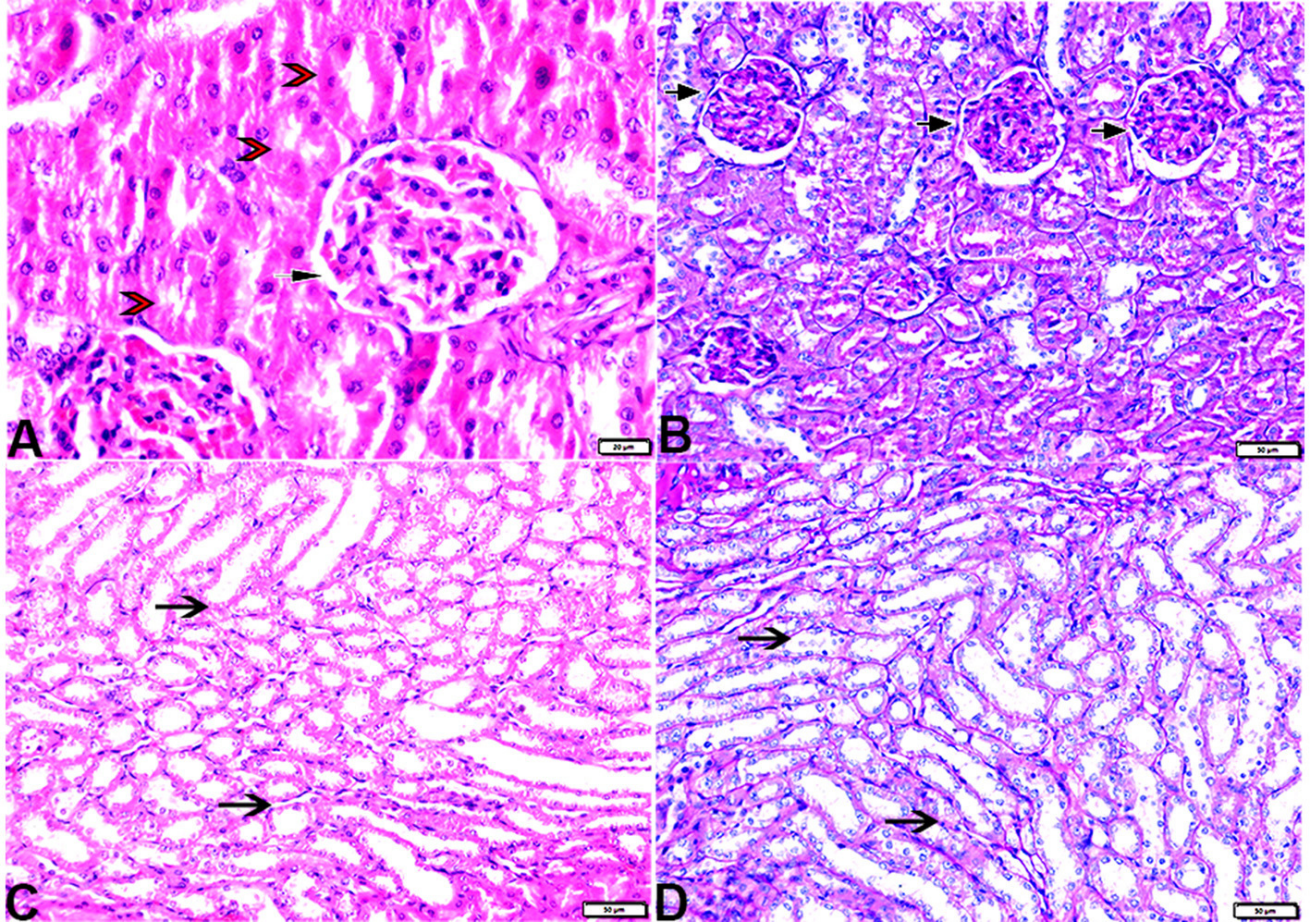
Photomicrograph of kidney tissue sections from control rats (G1): **(A)**: normal cortical structure compromising in normal glomerular size and structure (arrow) and normal renal tubules (arrowheads), **(B)**: normal glomerular basement membrane thickening (arrows), and **(C, D)**: normal renal medullary tubular structure (arrows). (A&C, H&E stain) (B&D, PAS). The bar size [**(A)** = 20 μm, **(B–D)** = 50 μm].

**Figure 9:**
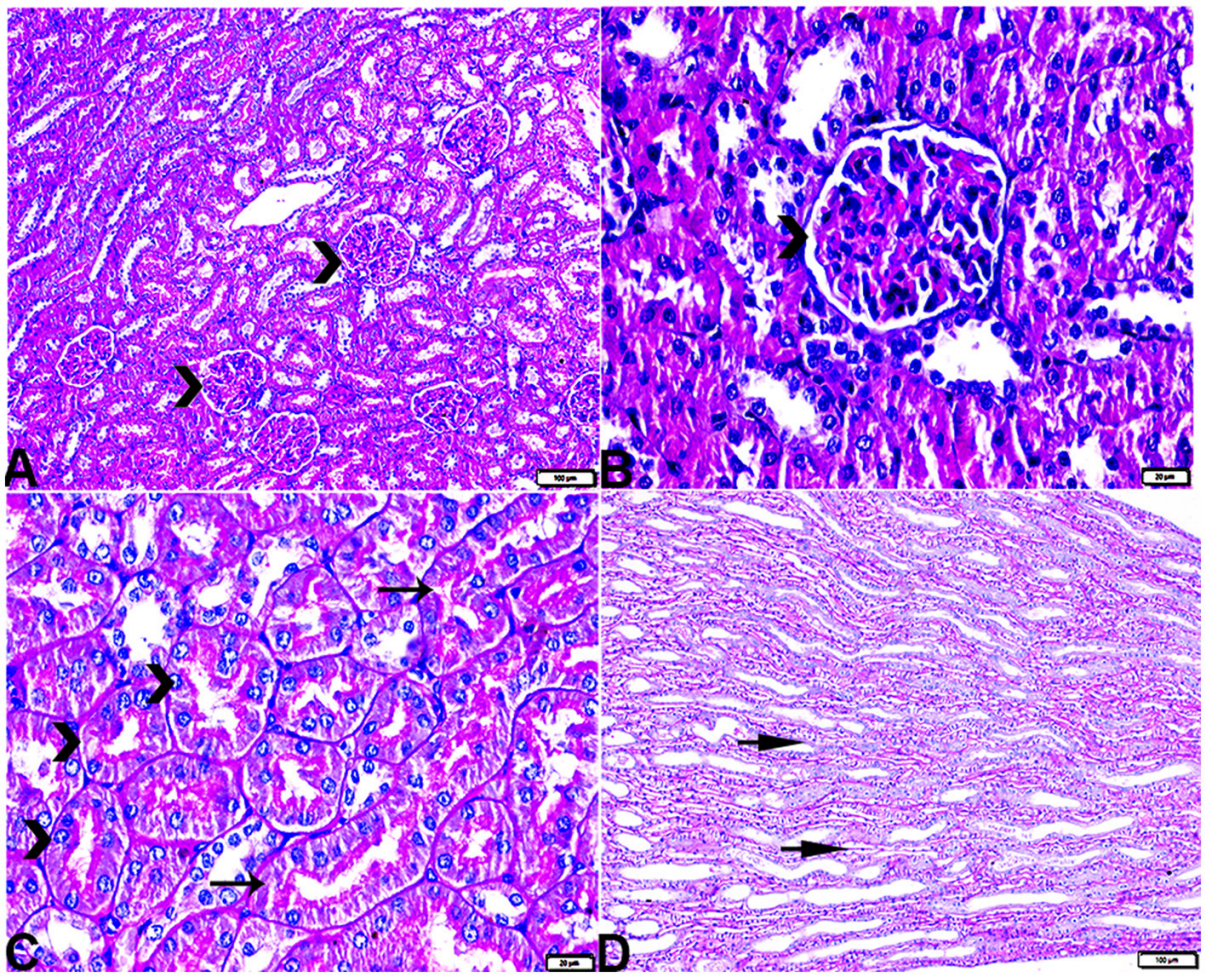
Photomicrograph of kidney tissue sections from melatonin control rats (G3): showing **(A)**: normal cortical structure compromising in normal glomerular size and structure (arrow), **(B)**: normal glomerular basement membrane thickening (arrows). **(C)**: normal proximal (arrowheads) and distal (arrows) convoluted tubules, **(D)**: normal renal medullary tubular structure (arrows). (A, H&E stains) (B, C&D, PAS). The bar size [**(A, D)** = 100 μm, **(B, C)** = 20 μm].

**Figure 10:**
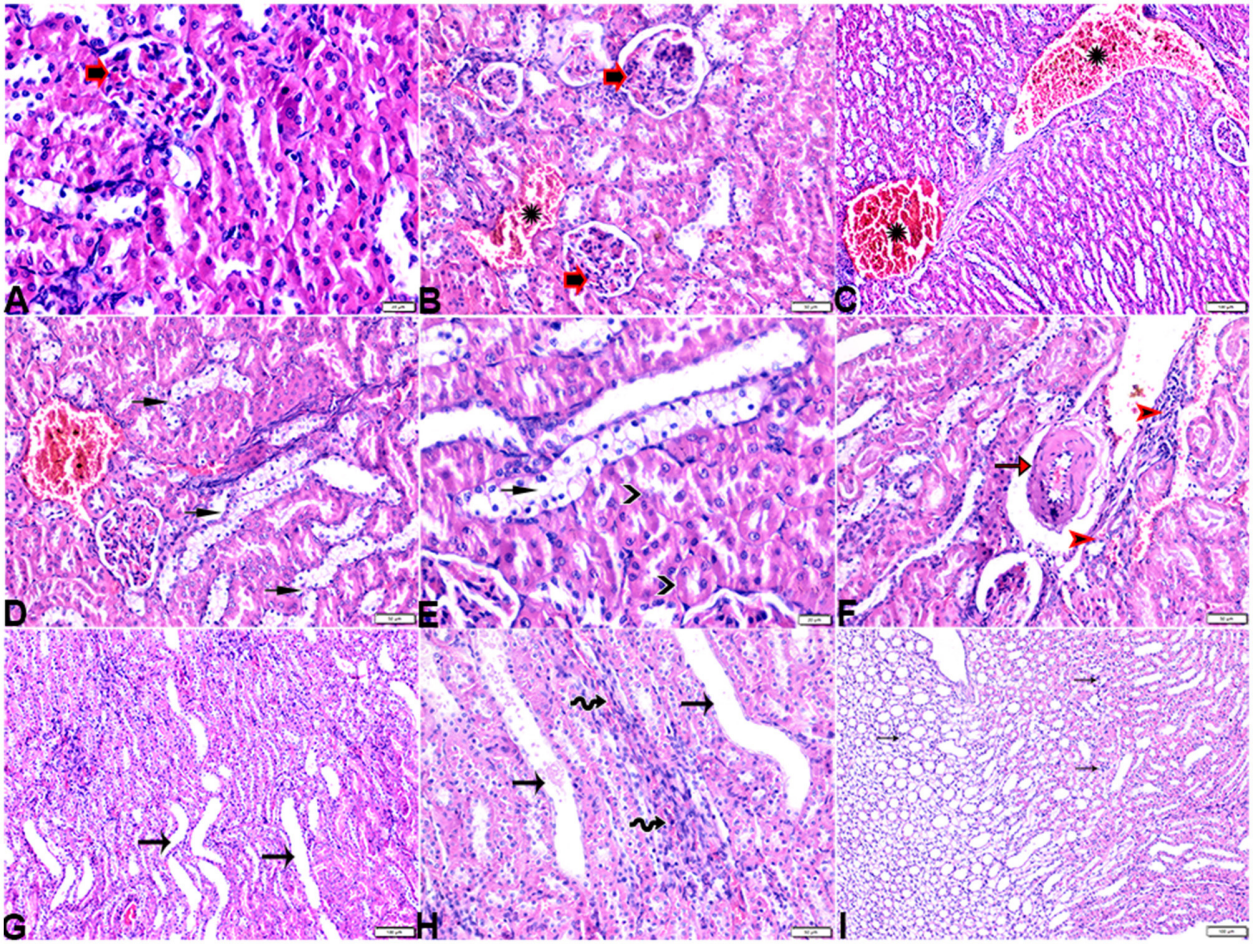
Photomicrograph of kidney tissue sections from diabetic untreated rats (G2): **(A–C)**: glomerular distortion with intraglomerular congestion (arrowheads), severe interstitial cortical congestion (stars), [**(D)** magnified in **(E)**]: vacuolar degeneration markedly in distal convoluted tubules (arrows), degeneration and desquamation in epithelium lining the proximal convoluted tubules (arrowheads), **(F)**: thickening in the arcuate artery at the corticomedullary junction (arrow) and fibrosis infiltrated with mononuclear cellular infiltration (arrowheads), and [**(G)** magnified in **(H)**]: medullary renal tubular dilatation (arrows) and interstitial fibrosis infiltrated with mononuclear inflammatory cells (zigzag arrows). Interstitial inflammatory cellular infiltration in between medullary renal tubules (arrows). H&E stain. The bar size [**(A)** = 20, **(B, D–F, H)** = 50 μm, **(C, G, I)** = 100 μm].

**Figure 11:**
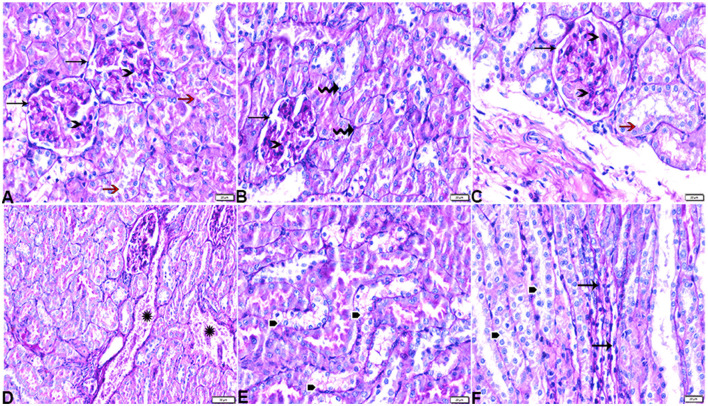
Photomicrograph of kidney tissue sections from diabetic untreated rats (G2): showing **(A–C)**: thickening in the glomerular basement membrane (membranous glomerulonephritis) (Bowman's capsule membrane) (arrows), thickening in the glomerular capillary membrane with expansion in mesangial cells (arrowheads), and thickening in the basement membrane lining renal tubules [**(B)** zigzag arrows]. Variable degenerative changes in cortical renal tubules [**(A, C)**, red arrows], **(D)**: cortical interstitial congestion (stars), **(E)**: severe vacuolar degeneration in epithelium lining distal convoluted tubules (arrowheads), and **(F)**: dilatation with vacuolar degeneration in epithelium lining medullary renal tubules (arrowheads) and interstitial fibrosis infiltrated with mononuclear inflammatory cells (arrows). PAS stain, the bar size [**(A–C, E, F)** = 20 μm and **(D)** = 50 μm].

The histopathological examination of kidney sections from the experimental groups demonstrated normal kidney histological structures in tissue sections obtained from rats of both the control (Figure 8) and MLT (Figure 9)—treated groups. Renal cortices revealed a normal glomerular size and structure and basement membrane thickening (Figures 8, 9A, B). Proximal and distal convoluted tubules showed typical cellular structures with normal thickening cellular outlines and a normal renal medullary tubular structure (Figures 8, 9C, D). Compared to the control, DM untreated rats exhibited degradation and deterioration in their glomeruli, thicker Bowman's capsule membrane, and congestion in their capillaries (Figures 10A, B). In the diabetic kidneys, the basal glomerulus membranes had thickened over their whole length with expansion in mesangial cells (Figures 11A–C). The tubular cells were enlarged with coarse pink granulocytes, particularly in the distal convoluted tubular cells, congestion in the interstitial tissue, and vacuolar degeneration in some tubular epithelial cells (Figures 10D, E, 11A, B, E). Nevertheless, some sinusoids were engorged with blood (Figures 10B–D). With regards to thickening in the arcuate artery at the corticomedullary junction, peri-arterial fibrosis was infiltrated with mononuclear inflammatory cells (Figure 10F). Medullary renal tubular dilatation, interstitial fibrosis infiltrated with mononuclear inflammatory cells were also noticed. In addition, interstitial inflammatory cellular infiltration in between medullary renal tubules was also observed in renal tissue sections from DM rats (Figures 10G–I, 11F).

**Figure 12:**
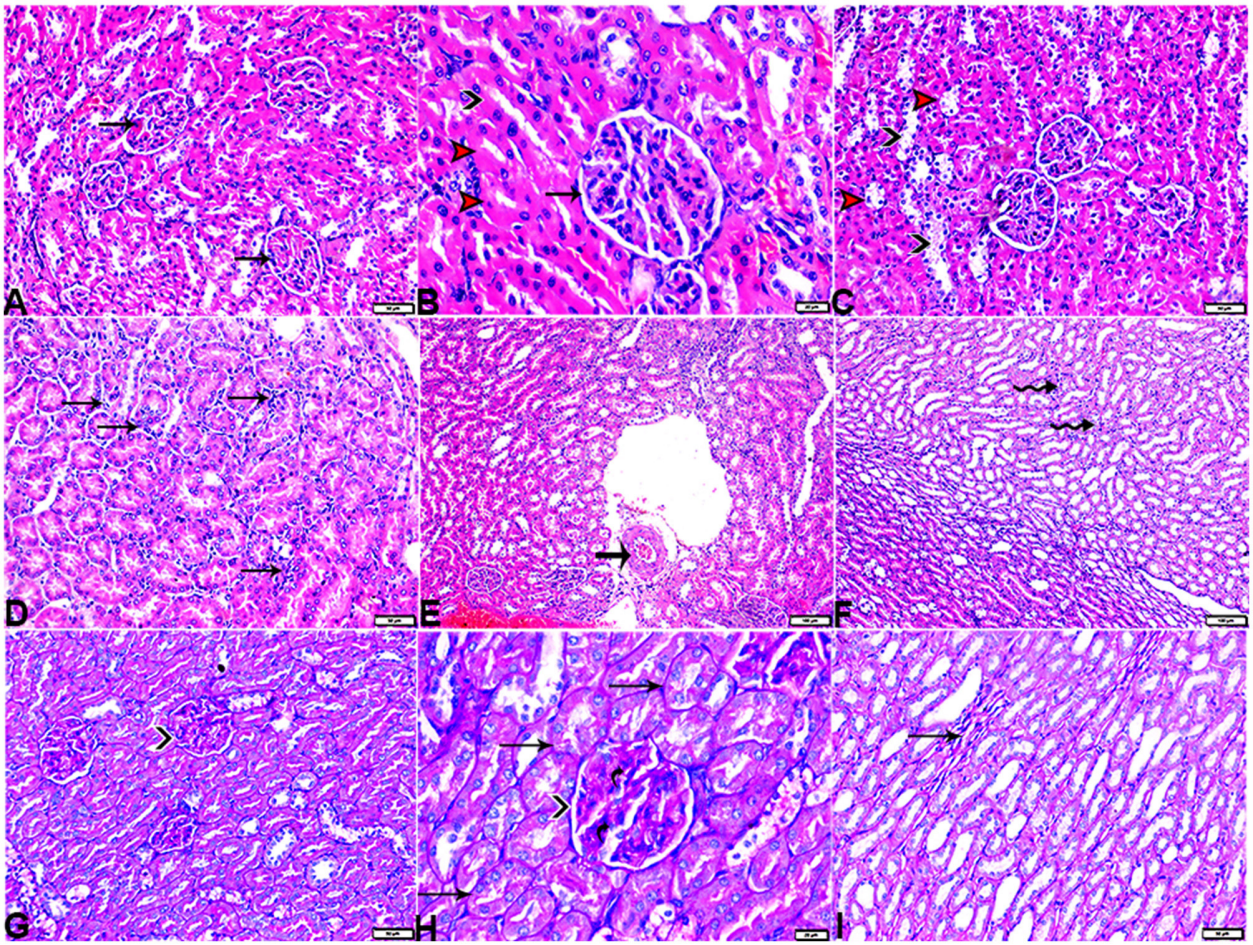
Photomicrograph of kidney tissue sections from diabetic rats treated with melatonin (G4): showing [**(A)** magnified in **(B)**]: normal glomerular size and structure (arrows), normal proximal (red arrowheads) and distal (black arrowhead), **(C)**: vacuolar degeneration in some proximal (red arrowheads) and distal (black arrowheads), **(D)**: interstitial mononuclear inflammatory cellular infiltration (arrows), **(E)**: normal arcuate artery at the corticomedullary junction (arrow), **(F)**: mild interstitial mononuclear inflammatory cellular infiltration (arrows), [**(G)** magnified in **(H)**]: mild thickening of the glomerular basement membrane (membranous glomerulonephritis) (Bowman's capsule membrane) (arrowheads) and mild expansion in mesangial cells (elbow arrows). Mild thickening in the basement membrane lining renal tubules (arrows), and **(I)**: mild interstitial fibrosis between medullary renal tubules (arrows). (A–F, H&E stain, G–I, PAS), the bar size [**(A, C, D, G, I)** = 50 μm, **(B, H)** =20 μm, and **(E, F)** = 100 μm].

**Figure 13:**
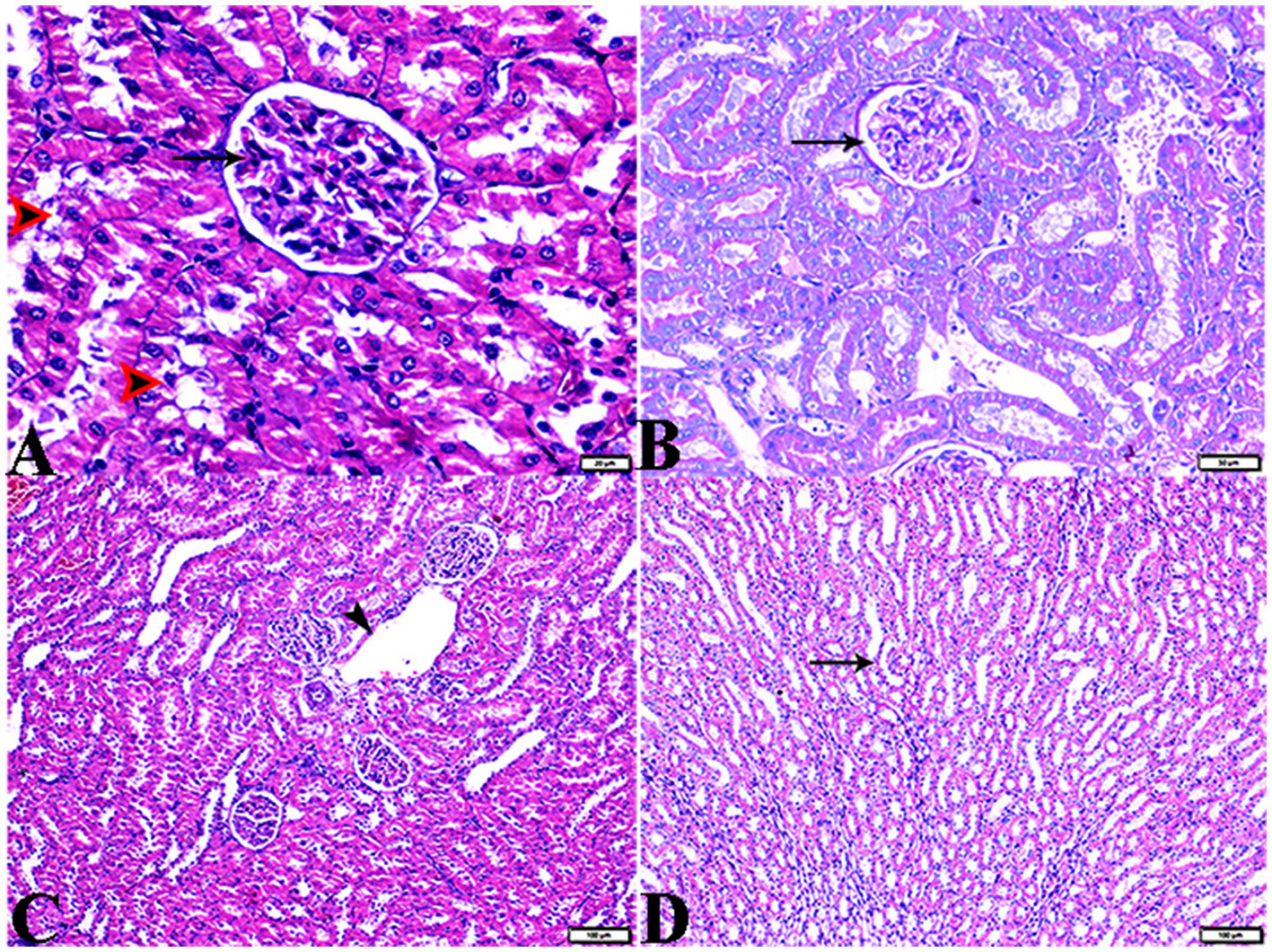
Photomicrograph of kidney tissue sections from diabetic rats treated with insulin (G5): **(A)**: a normal glomerular size with mild intraglomerular congestion (arrow) and mild vacuolar degeneration in cortical renal tubules (red arrowheads), **(B)**: mild thickening of the glomerular basement membrane (Bowman's capsule membrane) (arrow), **(C)**: normal arcuate artery at the corticomedullary junction (arrow), and **(D)**: normal renal medullary renal tubules (arrows). (A, C&D, H&E stain, B, PAS), the bar size [**(A)** = 20 μm, **(B)** = 50 μm, and **(C, D)** = 100 μm].

**Figure 14:**
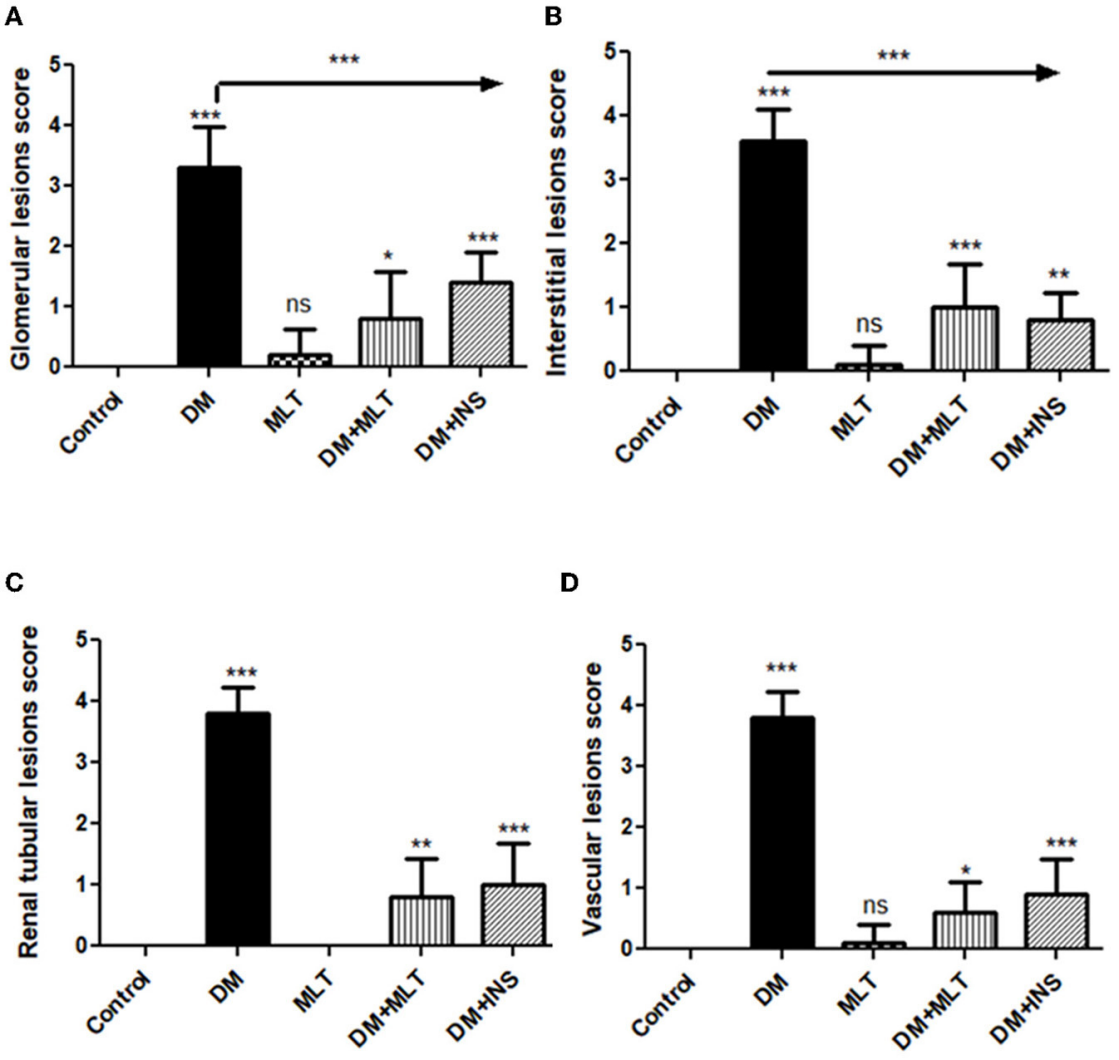
Histomorphometric graph showing semiquantitative measurements of lesion scores recorded in kidney tissue sections among the experimental groups: **(A)**: glomerular lesion score, **(B)**: interstitial lesion score, **(C)**: renal tubular lesion score, and **(D)**: vascular lesion score. Data are expressed as means ± standard deviations. Significant differences vs. the control group are marked by different asterisks through one-way ANOVA with Tukey's *post hoc* test: *p ≤ 0.05, **p ≤ 0.01, ***p ≤ 0.001.

In particular, these renal symptoms were alleviated in diabetic rats treated with melatonin (DM + MLT). Exogenous melatonin supplementation in diabetic rats resulted in significant glomeruli renewal and capillary integrity (Figures 12A–C, G, H). Even though a significant improvement was reported in this group (DM + MLT group), a slight histological change was noted upon examination of the renal tissues such as several tubules that appeared healthy, while others had vacuolar degeneration and cell debris in their lumens. A few proximal convoluted tubular cells were observed as vacuolated and swollen (Figures 12B, C). Numerous blood sinusoids were observed to be engorged with erythrocytes with interstitial congestion (Figure 12C), mild interstitial mononuclear inflammatory cellular infiltration (Figures 12D, F), arcuate artery at corticomedullary junction was normal (Figure 12E). In contrast, kidney tissue sections from the DM + INS group demonstrated a normal glomerular size with mild basement membrane thickening (mild membranous glomerulonephritis) and intraglomerular congestion (Figures 13A, B), and mild vacuolar degeneration in cortical renal tubules (Figure 13A). Normal arcuate artery at the corticomedullary junction (Figure 13C) and normal renal medullary renal tubules (Figure 13D). Histomorphometrically, the kidney tissue sections from the DM rats indicated significantly (*P* < 0.05) different types of cell damage in their tissue, which was characterized by glomerular alterations (Figure 14A), interstitial tissue changes, renal tubular degeneration, and vascular congestion (Figures 14B–D), as compared with other groups (Figures 14A–D). However, kidney tissue sections from DM + MLT rats showed a non-significant (*P* < 0.05) change as compared to control groups.

## 4. Discussion

Diabetes is believed to be a significant risk factor for cardiovascular disease (5), and therefore, it is not surprising to mention that diabetes has been classified as a coronary artery disease “risk equivalent” where hyperglycemia combines with a constellation of metabolic risk factors that accelerate atherogenesis (3). Despite extensive previous research on DM, only a few studies highlighted the association between hyperglycemia, glomerular alterations, and cardiovascular changes. In the present study, we sought to examine the serobiochemical alterations and explain their effects on the histopathological alterations in the heart and the kidney of diabetic animals before aggravating the chronic complications. The current data contain intriguing information on the potential role of melatonin in the restoration of several serobiochemical changes and restructuring of cardio-nephro diabetic vascular and cellular alterations in streptozotocin-treated rats. In the present study, DM rats showed a highly elevated serum TOC and blood glucose with a significant reduction in serum TAC and insulin as compared to the control group, whereas hyperglycemia generates free radicals through its role in the induction of lipid peroxidation with subsequent production of more reactive oxygen species. In addition, the elevated blood glucose level attenuates the antioxidative capacity in the patients through the non-enzymatic glycation of antioxidant enzymes (54, 55). In addition, hyperglycemia is associated with some cardiovascular complications, such as diabetic cardiomyopathy, in which cardiac fibrosis dominates due to the activation of cardiac fibroblasts into myofibroblasts that release extracellular matrix proteins, resulting in cardiac fibrosis (56). Furthermore, hyperosmolarity in hyperglycemia, shrinkage of ventricular cardiac myocytes, and an increase in collagen bundles may alter the size and shape of cardiomyocyte nuclei (57, 58).

Notably, CK-MB and myoglobin are considered as indicators for observing the effect of different glucose-lowering rates on myocardial damage. The CK-MB, a CK (creatine kinase) isoenzyme, is one of the best serum enzymes that are relied on as diagnostic indicators for myocardial infarction. Therefore, it has great specificity as a diagnostic procedure for myocardial injury (59). Taking this fact into account, myoglobin is the first non-enzyme protein for the identification of myocardial damage. It is regarded as one of the most precise diagnostic markers and a quick sensitive marker for coronary occlusion following recanalization (59). Data in the present study revealed a significant increase in serum CK-MB in the DM group as compared to the control group. This result is in in line with several previous reports (4, 60). Furthermore, the current data showed that elevated levels of AST and myoglobin were recorded in the DM group as compared to control rats. The present serobiochemical findings were confirmed histopathologically, whereas in DM rats, the structure of the heart tissues was disturbed by the presence of various histologic alterations in the cardiac tissues which suggested myocardial damage, as shown by the present study. However, the cardiomyocytes of the control group of rats were normal in size and orientation with a single, cigar-shaped centrally positioned nuclei. In the current study, heart-type fatty acid-binding protein (H-FABP) was significantly increased in the DM group as compared to the control group. The obtained result matches with those given in Otaki et al. (61) and Abir et al. (62). H-FABP is considered an important diagnostic biomarker for acute coronary syndrome and acute kidney damage (63) and its release into the blood circulation occurs after myocardial damage (49), and this obtained result was confirmed histopathologically where the heart tissue from DM rats showed various alterations that included cardiomyocyte death, vacuolation, hyalinization, shrinkage, a reduction in cardiomyocytes, and interstitial mononuclear inflammatory cell infiltration, which are characteristics of DM cardiac dysfunction, as demonstrated by a number of prior investigations (58, 64). Deformation of cardiomyocyte nuclei and disarray or disorganized cardiac myofibrils were also observed. In relation to tissue sections stained with Masson's trichrome, the connective tissue deposits were observed in the DM group compared to the control group, which indicated the presence of interstitial fibrosis in diabetic rats' cardiac tissue, which can affect both diastolic and systolic functioning (65). Previous studies identified diabetics with cardiomyopathy by diastolic or systolic failure and cardiac fibrosis (66).

In the present study, serum concentrations of endothelin-1 were highly elevated in DM rats, while the TNO levels were significantly decreased as compared to the control group. These results are in harmony with those of a previous study (67), which explained that endothelial dysfunction is an important characteristic of cardiovascular diseases. In addition, the imbalance of vasoconstrictors, such as endothelin-1, and a lower availability of vasodilator nitric oxide (NO) as a result of hyperglycemia-driven oxidative stress may lead to poor vasorelaxation, which plays a major role in microvascular and macrovascular problems of diabetes. Moreover, as the illness advances, the prolonged lack of protective NO effects and activation of the endothelin-1 system result in structural changes, thrombosis, and plaque formation in the arterial wall (67). Furthermore, nitric oxide is an important mediator for heart protection *via* its role as a substantial signaling molecule in the cardiovascular system where it is regarded as an endothelium-derived relaxing factor (68). In addition, hyperglycemia and lipid peroxidation reduce the NO content and bioavailability in the cells, which is necessary to prevent the superoxide anion production by the NADPH oxidase, leading to the accumulation of reactive oxygen species (ROS) (69). Moreover, the oxidative stress influence impairs the NO biosynthesis pathway, which strongly affects the vasodilation mechanism (70). Furthermore, endothelin-1 stimulates the proliferation of vascular smooth muscle cells (VSMCs) and promotes fibrosis and inflammation (67). Furthermore, cardiac fibrosis is an important step in the pathogenesis of diabetic cardiomyopathy, where various stimuli activate the cardiac fibroblasts (CFs) to myofibroblasts (MFs) that release extracellular matrix (ECM) proteins through the intracellular signaling pathway by transforming growth factor β1 (TGF-β1) which activates Smads-dependent signaling causing cardiac fibrosis in the hearts of diabetic mice (56).

The present study showed that the DM + MLT group showed a significant decrease in TOC, CK-MB, endothelin-1, Myogb, and H-FABP concentration as compared to the DM group. However, serum concentrations of TAC, TNO, and insulin in DM + MLT rats were significantly elevated in comparison with DM rats. This result could be related to the high antioxidant and anti-inflammatory characteristics of melatonin, which exhibited an outstanding preservation of the heart's histology and avoided heart fibrosis, demonstrating an anti-fibrotic impact of MLT (12). Our findings agree with those of the previous study (56) and revealed that melatonin produces an antifibrotic effect through the inhibition of lncR-MALAT1/miR-141-mediated NLRP3 inflammasome activation and TGF-β1/Smads signaling. Additionally, some reports indicated that MLT maintains the integrity of cardiomyocytes and inhibits membrane degradation (71). In addition, the therapy with MLT considerably reduced the elevated levels of AST and ALT in the present study. These results are comparable with those of several prior research studies (72, 73) that reflected the efficacy and safe application of melatonin.

In the current study, both urea and creatinine were significantly elevated in the DM group in comparison to control rats. These results match those given in a previous study (55), and these findings were confirmed by our histopathological results. It is important to emphasize that diabetic-reliant nephropathy is distinguished by kidney morphological changes (74). Nevertheless, according to our investigations on DM rats, glomerulus membranes thickened (membranous glomerulonephritis) with expansion in mesangial cells, congestion in the interstitial tissue, and vacuolar degeneration in some tubular epithelial cells. Thickening in the arcuate artery at the corticomedullary junction, peri-arterial fibrosis infiltrated with mononuclear inflammatory cells. Those features were observed in addition to dilatation in medullary renal tubules. Our findings agree with the findings of a previous study (75) which reported that variations in mesangial matrix and basement membrane thickness have a strong association with nephropathy. Mesangial expansion induces the collapse of a part and then of all of the capillary lumens, resulting in a significant increase in glomerular size (75). Furthermore, the release of fibronectin, collagen IV, and laminin may contribute to the thickening of basement membranes and interstitial fibrosis (75).

Notably, early histological changes that occur in the diabetic kidney may include tubular basement membrane thickening and inflammation of the interstices with mononuclear cell infiltration. Tubulointerstitial abnormalities lead to tubulointerstitial fibrosis as they develop. Diabetes mellitus can influence renal arteries of any size (76, 77). Hyalinosis develops in both afferent and efferent arterioles in diabetic nephropathy, although the engagement of efferent arterioles is more specific (78). Despite the fact that many of these findings corroborate this idea, a few of them have also shown that hyperfiltration has been linked to vascular hypertrophy, vasodilation, and injury to the glomerular and tubular arteries (77). It was hypothesized that regulating blood pressure and controlling hyperglycemia are the most effective means of preventing microvascular damage and additional issues in the diabetic kidneys (79). It is a gradual consequence of the diabetic kidneys that induce hypertension and ischemic nephropathy. During these cellular activities, diabetic people release enormous numbers of reactive oxygen species, nitrogen species, and other free radicals (80). Consequently, oxidative stress plays a crucial role in the reported renal impairment. Intriguingly, the current study indicated that melatonin treatment led to significant changes in renal tissue structures, which was consistent with the result that melatonin administration ameliorates experimentally caused acute kidney damage (81).

## 5. Conclusion

Given the above information, melatonin has been shown to ameliorate a series of serobiochemical parameters that include blood glucose, insulin, TAC, TOC, serum concentration of TNO, CK-MB, endothelin-1, myoglobin, H-FABP, ALT, and AST. In addition, melatonin efficiently restored the histopathological alterations observed in diabetic DM rats to the near-normal structure. Melatonin's hypoglycemic, anti-inflammatory, and antioxidant characteristics make it potentially effective in preventing diabetic heart and renal damage. In this study, the capacity of melatonin to regulate serobiochemical markers and reduce histopathological alterations was observed. The present study suggested the potential use of melatonin as a promising therapeutic target for diabetic cardio-nephropathy.

## Data availability statement

The original contributions presented in the study are included in the article/supplementary material, further inquiries can be directed to the corresponding authors.

## Ethics statement

This study and all experimental procedures were performed according to the principles of the Ethics Committee of Taif University, Taif, Saudi Arabia (Approval No. HAO-02-T-105) which are in line with the Declaration of Helsinki.

## Author contributions

NN contributed to the analysis of serum biochemical parameters, also did the statistical analysis of the results of serum biochemical parameters, prepared the tables, wrote the original manuscript draft and contributed to the sampling, revised the final manuscript, and contributed to sending a response to the reviewers. FA contributed to experimental design, sampling, and histopathological and morphometrical assessments, wrote the manuscript draft, revised the final manuscript, and contributed to sending a response to the reviewers. EE, KA, MA, AH, and WA were involved in the conception of the idea and methodology design, and prepared the manuscript for publication and revision. AMA, AO, OA-A, AJAA, ML, and AAl were involved in methodology design, performed data analysis and interpretation, and prepared the manuscript for publication. All authors read and approved the final manuscript.
