# Corrigendum: Melatonin ameliorates serobiochemical alterations and restores the cardio-nephro diabetic vascular and cellular alterations in streptozotocin-induced diabetic rats

**DOI:** 10.3389/fvets.2023.1240320

**Published:** 2023-07-18

**Authors:** Khalaf F. Alsharif, Ehab Kotb Elmahallawy, Mohamed A. Alblihd, Asmaa A. Hamad, Nani Nasreldin, Walaa Alsanie, Ahmad Majed Aljoudi, Atif Abdulwahab A. Oyouni, Osama M. Al-Amer, Alaa Jameel A. Albarakati, Maha S. Lokman, Ashraf Albrakati, Fatma Abo Zakaib Ali

**Affiliations:** ^1^Department of Clinical Laboratories Sciences, College of Applied Medical Sciences, Taif University, Taif, Saudi Arabia; ^2^High Altitude Research Center, Taif University, Taif, Saudi Arabia; ^3^Department of Zoonotic Diseases, Faculty of Veterinary Medicine, Sohag University, Sohag, Egypt; ^4^Department of Medical Microbiology and Immunology, College of Medicine, Taif University, Taif, Saudi Arabia; ^5^Department of Biology, College of Science, Taif University, Taif, Saudi Arabia; ^6^Department of Pathology and Clinical Pathology, Faculty of Veterinary Medicine, New Valley University, El-Kharga, Egypt; ^7^Hera General Hospital, Makkah, Saudi Arabia; ^8^Department of Biology, Faculty of Sciences, University of Tabuk, Tabuk, Saudi Arabia; ^9^Genome and Biotechnology Unit, Faculty of Sciences, University of Tabuk, Tabuk, Saudi Arabia; ^10^Department of Medical Laboratory Technology, Faculty of Applied Medical Sciences, University of Tabuk, Tabuk, Saudi Arabia; ^11^Department of Surgery, College of Medicine, Umm Al Qura University, Makkah, Saudi Arabia; ^12^Department of Biology, College of Science and Humanities in Al-Kharj, Prince Sattam Bin Abdulaziz University, Al-Kharj, Saudi Arabia; ^13^Department of Zoology and Entomology, Faculty of Science, Helwan University, Cairo, Egypt; ^14^Department of Human Anatomy, College of Medicine, Taif University, Taif, Saudi Arabia; ^15^Department of Pathology and Clinical Pathology, Faculty of Veterinary Medicine, Sohag University, Sohag, Egypt

**Keywords:** melatonin, TNO, endothelin-1, H-FABP, heart, kidney

In the published article, there was an error regarding the author list. The first author in the list should have been Khalaf F. Alsharif and the second author should have been Ehab Ktob Elmahallawy. Additionally, the † symbol should have been included to indicate that both of these authors share first authorship.

In the published article, there was an error regarding the affiliation(s) for Khalaf F. Alsharif^1^, Mohamed A. Alblihd^4^ and Asmaa A. Hamad^5^. As well as having affiliation(s) 1, 4 and 5 respectively, they should also have ^2^High Altitude Research Center, Taif University, Taif, Saudi Arabia.

Khalaf F. Alsharif^1,2^, Mohamed A. Alblihd^2,4^ and Asmaa A. Hamad^2,5^

^2^High Altitude Research Center, Taif University, Taif, Saudi Arabia

The authors apologize for this error and state that this does not change the scientific conclusions of the article in any way. The original article has been updated.

